# Complete Genome Sequence of the Type Strain *Corynebacterium mustelae* DSM 45274, Isolated from Various Tissues of a Male Ferret with Lethal Sepsis

**DOI:** 10.1128/genomeA.01012-15

**Published:** 2015-09-10

**Authors:** Christian Rückert, Janine Eimer, Anika Winkler, Andreas Tauch

**Affiliations:** a Department of Biology, Massachusetts Institute of Technology (MIT), Cambridge, Massachusetts, USA; b Institut für Genomforschung und Systembiologie, Centrum für Biotechnologie (CeBiTec), Universität Bielefeld, Bielefeld, Germany

## Abstract

The complete genome of *Corynebacterium mustelae* DSM 45274 comprises 3,474,226 bp and 3,188 genes. Prominent niche and virulence factors are SpaBCA- and SpaDEF-type pili with similarity to pilus proteins of *Corynebacterium resistens* and *Corynebacterium urealyticum* and an immunomodulatory EndoS-like endoglycosidase probably catalyzing the removal of distinct glycans from IgG antibodies.

## GENOME ANNOUNCEMENT

The type strain of the species *Corynebacterium mustelae*, DSM 45274 (3105^T^), was originally cultured from necropsy lung tissue, the liver, and the kidneys of a male ferret with lethal sepsis (1). Biochemical reactions, chemotaxonomic features, and sequencing of the 16S rRNA gene provided clear evidence that strain 3105^T^ is a representative of a novel *Corynebacterium* species. It can be distinguished from all other corynebacteria by a “humid cellar-like” odor, strong adherence to agar, and the synthesis of a greenish-beige pigment (1, 2). *C. mustelae* was also isolated from a blood culture of a Munsterlander dog with ventricular septal defect (3) and was detected as an operational taxonomic unit in a study of the canine oral microbiome (GenBank accession numbers KF030214 and KF030215). However, transmission of *C. mustelae* from animals to humans, for instance, by a dog bite (4–7), has not been observed so far. Here, we present the genome sequence of *C. mustelae* DSM 45274 to provide genetic data for a corynebacterium that has been isolated from animals with severe disease.

Genomic DNA of *C. mustelae* DSM 45274 was obtained from the Leibniz Institute DSMZ. A whole-genome shotgun library was constructed with the TrueSeq DNA PCR-free library preparation kit (Illumina) and was sequenced in a paired-end run using the MiSeq reagent kit v3 (600 cycles) and the MiSeq desktop sequencer (Illumina). Shotgun sequencing generated 4,617,899 paired reads with 657,639,232 detected bases. The paired reads were assembled with the Roche GS *de novo* Assembler software (release 2.8), resulting in 24 scaffolds and 55 scaffolded contigs. A 7-kb mate pair library was prepared and sequenced with the Nextera mate pair sample preparation kit (Illumina) and the MiSeq reagent kit v3 (600 cycles). Mate pair sequencing generated 126,936 reads that were added to the initial genome assembly. Remaining gaps in the genome sequence were closed *in silico* with the Consed software (version 26) (8). The regional gene prediction was performed with Prodigal (9) and the functional annotation of the predicted coding regions was carried out by the IMG/ER software (10).

The genome of *C. mustelae* DSM 45274 consists of the bacterial chromosome with a size of 3,391,554 bp and a mean G+C content of 52.2%, plasmid pCMUS45274 (39,867 bp with 51.3% G+C content), and corynephage ΦCMUS45274 (42,805 bp with 56.4% G+C content). The annotation of the genome sequence revealed 3,094 chromosomal genes, 40 genes on pCMUS45274, and 54 genes on phage ΦCMUS45274. The predicted extracellular proteome of *C. mustelae* DSM 45274 includes two types of adhesive pili (11) showing similarity to the SpaABC pilus of *Corynebacterium resistens* DSM 45100 (12) and to the SpaDEF pilus of *Corynebacterium urealyticum* DSM 7109 (13). Moreover, *C. mustelae* contains an *ndoS* gene encoding a secreted endoglycosidase of the EndoS family that is probably able to remove the N-linked glycans from the chitobiose core of IgG antibodies. This removal results in the inability of IgG to bind to antibody receptors of white blood cells and in a significant loss of function, increasing the bacterial survival in the host’s blood (14, 15).

### Nucleotide sequence accession numbers. 

This genome project has been deposited in the GenBank database under the accession numbers CP011542 (*C. mustelae* DSM 45274 chromosome), CP011543 (pCMUS45274), and CP011544 (ΦCMUS45274).
